# Computational assessment of long-term memory structures from SDA-M related to action sequences

**DOI:** 10.1371/journal.pone.0212414

**Published:** 2019-02-22

**Authors:** Benjamin Strenge, Ludwig Vogel, Thomas Schack

**Affiliations:** 1 CITEC – Center of Excellence “Cognitive Interaction Technology”, Bielefeld University, Bielefeld, Germany; 2 Neurocognition and Action Research Group, Bielefeld University, Bielefeld, Germany; Educational Testing Service, UNITED STATES

## Abstract

Assistance systems should be able to adapt to individual task-related skills and knowledge. Structural-dimensional analysis of mental representations (SDA-M) is an established method for retrieving human memory structures related to specific activities. For this purpose, SDA-M involves a semi-automatized survey of users (the “split procedure”), which yields data about users’ associations between action representations in long-term memory. Up to now this data about associations has commonly been clustered and visualized by SDA-M software in the form of dendrograms that can be used by human experts as a tool to (manually) assess users’ individual expertise and identify potential issues with respect to predefined action sequences. This article presents new algorithmic approaches for automatizing the process of assessing task-related memory structures based on SDA-M data to predict probable errors in action sequences. This automation enables direct integration into technical systems, e.g. user-adaptive assistance systems. An evaluation study has compared the automatized computational assessments to predictions made by human scholars based on visualizations of SDA-M data. The different algorithms’ outputs matched human experts’ manual assessments in 84% to 86% of the test cases.

## 1 Introduction

Suitable prediction of human behavior is a highly promising, but also challenging objective [1]. Predictions about a specific person’s memory lapses and action errors with respect to given tasks can not only help human teachers or trainers to focus their instruction on each trainee’s weak points, but could also be fed into a wide spectrum of technical assistance systems to support user-specific adaptation. This is especially important when subsystems of human cognition with limited capacity, such as those related to attention, are required to deal with different sources of input in parallel. Our research focuses on investigating how anticipatory assistance systems can facilitate cognitive aspects of human activities and human-machine interaction. Prototypical application scenarios for this are in-car driver information systems and assistive smart glasses overlaying the real world with virtual content. For example, the recently researched ADAMAAS smart glasses are intended to assist disabled or elderly people in daily activities [2]. In such contexts, giving excessive step-by-step assistance for a task by constantly placing vast amounts of visual information within the users’ field of view could be annoying and distracting at best, perhaps even dangerous. It may also lead to a high degree of dependence on the technical systems and impede learning processes when users resort to mindlessly following a system’s instructions. Instead, the amount of information presented to users should be restricted to the required minimum. This generally conforms with established principles from disciplines such as human-centred design (ISO 9241-110; ISO 14915), human-computer interaction [3], ergonomics (ISO 15005), and usability engineering [4]. Therefore, it must be determined in which situations assistance is actually required. This is the case when users are either unsure about what to do, or when they would do something wrong. In perilous or time-critical task sequences, these situations should obviously be anticipated beforehand to mitigate possible damage. In non-critical activities, feasible predictions can contribute to smoother task execution, better user experience and better performance rather than waiting for human errors to occur and trying to correct them afterwards. Technical systems that incorporate such an anticipatory module, combined with effective assistance features, can induce a new level of learning processes.

This article proposes a new computational approach for generating such predictions. To this end, task-related knowledge in each individual’s long-term memory is retrieved using *structural-dimensional analysis of mental representations* (SDA-M) [5]. A software-based prediction module is then used to assess individual problems in task execution. Finally, we report results from a study assessing the expected accuracy of different alternative algorithmic approaches.

## 2 Task-related mental representation structures

From a cognitive-perceptual perspective mental representations can be considered as the cognitive basis to organize and execute complex motor actions and movements [6]. In the middle of the 19th century, classical ideas in psychology led to the “ideomotor” approach [7, 8], which distinguishes the important role of a cognitive equivalent of actions in memory [9]. Since that time different lines of research in cognitive psychology, philosophy, cognitive robotics and other disciplines refer to the central role of mental representations in action organization with different definitions and perspectives [10–13]. For the purpose of this article it seems useful to refer to mental representations as a functional structure that integrates both perceptual and cognitive features to achieve context-specific action goals [12].

### 2.1 Background

Planning and acting in a goal-oriented way requires a structured cognitive basis that integrates person, environment, and task information [14–16]. Cognitive aggregations and chunking reduce the planning cost and facilitate action and movement control [17, 18]. From this point of view, mental representations overcome the complexity of redundant environments to control complex movements and action sequences, leading to task-related order formation. A seminal theoretical framework for movement control by Bernstein [19] described the multiple ways to reach a movement goal as a degrees-of-freedom problem. Bernstein developed a task-dependent evolutionarily-originated multi-level model of movement control. However, the idea of a hierarchical cognitive architecture has been investigated using diverse approaches [20–23]. A suitable model for the research presented in this article was proposed in [24]. The model of the cognitive architecture of action uses a goal-oriented approach of regulatory levels and representational levels that are functionally autonomous [13]. This so-called “cognitive action architecture approach” (CAA-A) differentiates between two regulatory levels of mental control (level IV) and sensorimotor control (level I) which initiate volitional and control strategies (IV) and lower level processes, such as automatized movements and reflexes (I), respectively [25, 26]. The two representational levels of sensorimotor (II) and mental representation (III) build the cognitive information basis. Whereas perceptual effects and their spatial-temporal features are stored on the sensorimotor representation level (II), the cognitive units of complex actions, the so-called basic action concepts (BACs), are located on the level of mental representation (III). BACs can be seen as the building blocks of motor memory that are connected to perceptual effects of actions [12, 13]. A number of studies have investigated the essential role of BACs in long-term memory in manual actions [27], sports actions [6, 28], sports tactics [29] or rehabilitation [30, 31]. The results characteristically show that mental representations of people with a high level of competence and expertise are well-integrated tree-like structures that are in line with the biomechanical structure of the task. However, the mental representations of novices, young children or stroke patients reveal less hierarchically organized cognitive structures. These findings are supported by experiments from [25] regarding modularity in motor control [32] which indicated a clear structural relationship between mental representation and the kinematic structure of movement. Furthermore, current projects and investigation on job-related knowledge have been conducted [13, 33, 34]. We assume that, as for tactical knowledge and complex actions, the structures of working tasks in occupational rehabilitation are stored in memory [29] and change over the course of learning [35]. The cognitive representation of such tasks can be investigated by applying the structural-dimensional analysis of mental representations (SDA-M) method.

### 2.2 Analysis with the SDA-M method

With the SDA-M it is possible to analyze human memory structures related to a given set of items (e.g. actions). We argue that well-integrated cognitive networks lead to structured decisions in the SDA-M split procedure. The method then provides psychometric data that can be analyzed on an individual and on a group level. To this end, SDA-M can comprise up to four phases [5, 36] which are outlined in the following.

#### 2.2.1 Step 1: Split procedure and distance scaling

**Fig 1:**
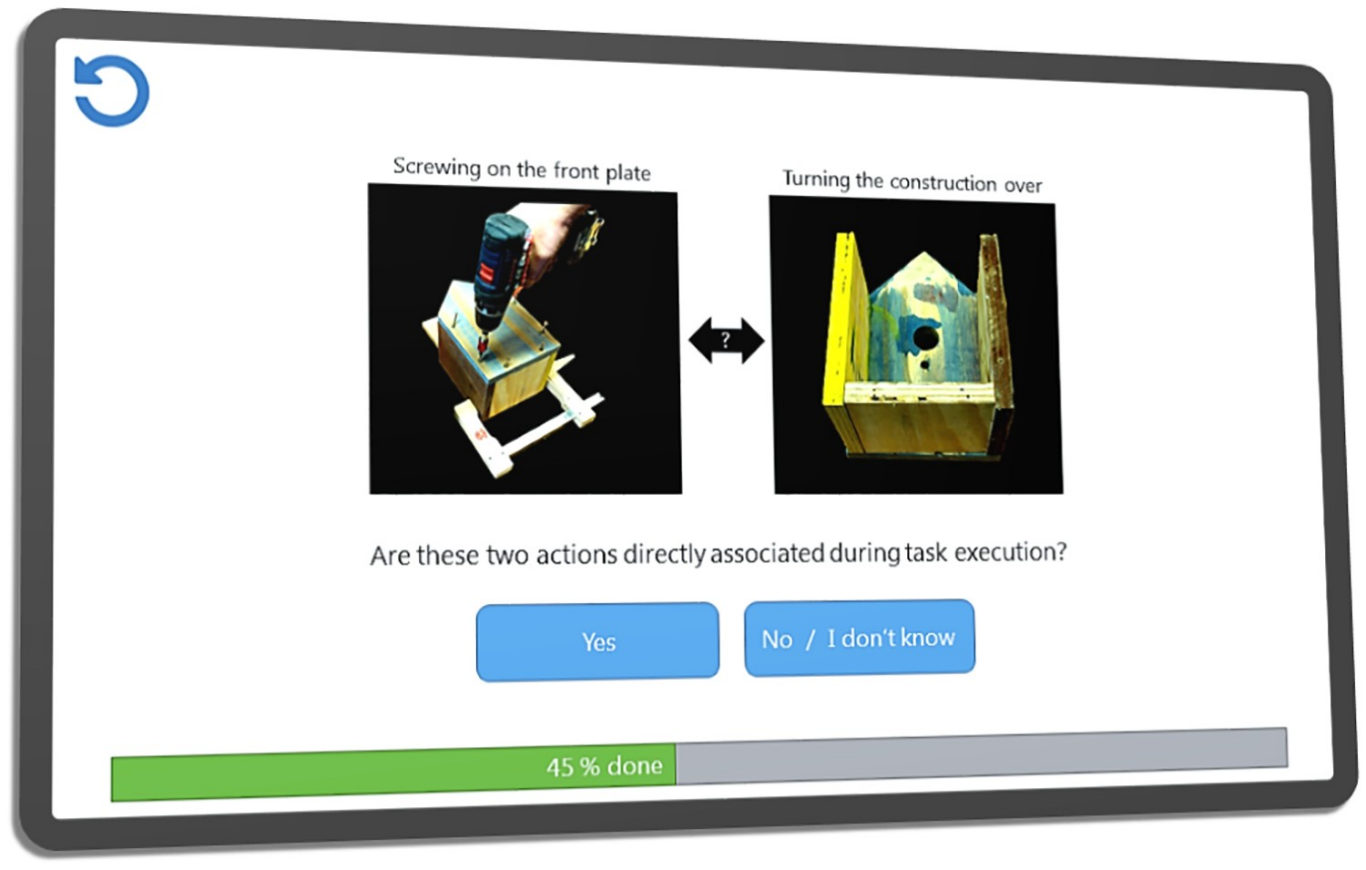
SDA-M software interface. In this UI concept illustration of our SDA-M tool for mobile devices the two exemplary action representations are related to the activity ‘building a birdhouse’.

When SDA-M is used to analyze a specific task or activity, the task/activity is first split into sub-tasks or actions which are indicated by textual descriptions, pictures or illustrations, short video clips, or a combination of those means. This is done by researchers in collaboration with domain experts (e.g. coaches) in order to provide a “plausible and workable set” of actions [5]. These action items are then shown to study participants or users on a computer screen. Fig 1 shows the split procedure user interface concept for the mobile touch-friendly version of our SDA-M software. Actions are chosen in random order as reference objects or “targets” and then, one after another, all other actions are compared to the current target in random order. The user must decide for each pair of actions whether these are directly associated during task execution or not. The decisions made in the context of each target result in a particular decision tree, i.e. in the end the number of decision trees is equal to the total number of actions. The tree for a given target consists of nodes containing the subsets of actions which the user considered as “associated” or “not associated” to the target in a splitting step. Hereby a value of *x*_*i*_ = *s*_*i*_ * |*N*_*i*_| is assigned to each action, with |*N*_*i*_| being the number of actions contained in the node; *s*_*i*_ = 1 if the user stated that the actions in the node were “associated” to the target, and *s*_*i*_ = −1 otherwise. (Note: Multiple splitting steps may be performed for each reference action in order to yield a more fine-grained distance measure. However, most contemporary applications of SDA-M, including this study, are restricted to only one splitting step for each reference action, resulting in binary decision trees of height 1, in order to reduce the required time and effort for participants.) [37] argued that a metrically defined measure of distance from a reference object (target) to any other can be obtained by standardizing the respective *x*_*i*_ values to *z*-scores, thus establishing a “*Z* matrix” containing one such (row) vector of *z*-scores for each action. The SDA-M software then creates matrices containing the correlations (“*R* matrix”) and Euclidean distances (“*D* matrix”) between all rows of the *Z* matrix. The distance values in the *D* matrix (or, equivalently, the correlation values in the *R* matrix) contain all information to completely define an individual’s representational structure [38]. The subsequent steps of SDA-M are therefore functions of these matrices.

#### 2.2.2 Step 2: Hierarchical clustering and visualization

The distances calculated in the first step are now used as the metric for hierarchical agglomerative average-linkage clustering. The results are visualized by a dendrogram to facilitate human assessment of the mental representation structure. Many SDA-M applications stop at this point.

#### 2.2.3 Step 3: Extraction of feature dimensions

The third step aims to uncover the latent criteria or feature dimensions that seem to have guided subjects’ decisions during the split procedure. To this end, the R matrix is subjected to factor analysis with a special cluster-oriented rotation procedure [5, 36], the details of which are irrelevant for this article.

#### 2.2.4 Step 4: Analysis of interindividual differences

Finally, pairs of individual or subgroup-specific cluster structures can be analyzed for their degree of similarity. SDA-M employs the invariance measure λ for this purpose, which takes the total numbers of clusters and their pairwise congruence into account.

#### 2.2.5 Usage in individual cognitive assessment and coaching

Numerous previous studies have indicated that educated psychologists and domain experts could use dendrograms from SDA-M (step 2) to detect individual issues regarding action execution and derive helpful advice for performance optimization, e.g. [16, 24, 39–41]. The SDA-M method enables addressing individual needs by taking the essential information about the underlying cognitive-perceptual action system into account [16]. For example, mental representations related to gymnastics skills were retrieved from novices and experts. Individual mistakes in carrying out the movement were analyzed based on SDA-M data. It was reported that individual interventions based on those mental representations accelerated and optimized the learning process and brought novices’ mental representation structures closer to those of experts [26, 35, 39]. The SDA-M method has been applied to numerous activities in manual action, sports, dancing and rehabilitation [13, 24, 26, 28, 42] to investigate expertise-dependent memory structures and develop related individualized training strategies [16, 43].

## 3 Algorithmic prediction of human action errors

With reference to the abovementioned research publications we take for granted that SDA-M data visualized as dendrograms can be interpreted by appropriately trained human specialists (psychologists, mathematicians etc.) to identify deficits in memory structures. On account of this, the current study investigated how SDA-M data can be *automatically* interpreted by a technical system and trigger corresponding assistance when needed. We developed two alternative algorithmic approaches to human error prediction based on SDA-M data. These shall be called *Analysis of Most Probable Actions* (AMPA), and *Correct Action Selection Probability Analysis* (CASPA), respectively. Both algorithmic approaches require as input

- a predefined list of all correct action sequences (related to an activity), and
- valid SDA-M data for a specific person X (related to an activity).

The output of the algorithms then indicates when (i.e. after which actions) person X may require assistance while performing the activity.

### 3.1 Assumptions and prerequisites

Both algorithms require the overarching activity or task to be represented in SDA-M through a set of *n* subtasks or actions *(“BACs”)* satisfying the following criteria:

- *Atomicity*: Each action is self-contained insofar as it is assumed to be executable by each person without issues. If this was not the case, it must be divided further into feasible sub-actions before performing the SDA-M split procedure. The resulting BACs can be understood as problem-solving operators available to users.
- *Sequential discreteness*: Actions do not overlap in time. All correct sequences of actions can be formed by strictly ordering a subset of all actions.
- *Non-recurrence*: Each action appears at most once in each correct action sequence. (Note: In practical applications this restriction can often be worked around by adding sequential information to descriptions of identical actions in the SDA-M split procedure, e.g. *“Pressing the yellow button for the first time*” and *“Pressing the yellow button for the second time*”).
- *Completeness*: The total set of actions considered during the SDA-M split procedure comprises all actions that can be executed while performing the activity.
- *Context-independence*: Environmental and contextual factors not explicitly incorporated into action descriptions do not influence behavior.
- *Currentness*: The SDA-M data for a given person is valid in the sense that his or her task-related memory structure has not changed since the SDA-M split procedure was performed.

In practical applications these theoretical assumptions may not hold to full extent, hence decreasing the achievable accuracy of predictions, but not necessarily rendering the results unusable. For example, the assumption of *completeness* will commonly be violated to some degree by focusing, for pragmatic reasons, on a set of *probable* task-related actions instead of all *possible* actions. This is inevitable because the SDA-M split procedure (the “manual” part of the method) has a time complexity of Θ(*n*^2^), i.e. the time for performing it grows quadratically as a function of the number of actions. According to our experience in practice this usually limits the number of incorporable actions to approximately 10-15 (depending on the time required for each decision), because subjects are rarely willing to perform split procedures lasting much longer than quarter of an hour. In a similar vein [38] stated that the number of actions “should not be chosen higher than 20. Otherwise, the decisions made regarding the similarity of stimuli may become inconsistent”. The requirement of sequential discreteness must be accounted for when determining the actions (“BACs”). Furthermore, participants should be disposed to ideally associate each action exactly with what they believe to be the immediate preceding and subsequent actions with respect to correct sequences. To this end, the current version of our SDA-M software incorporates an introductory video (in German) that instructs participants to state whether the displayed actions are executed immediately before or after another during task execution. Note that many, but not all previous applications of SDA-M complied with the requirement of sequential discreteness [30, 31, 43].

### 3.2 Algorithm I: Analysis of Most Probable Actions (AMPA)

The first step of SDA-M involves calculating a measure of distances between any two of the analyzed items (e.g. objects or actions) in a person’s long-term memory. Algorithm I determines whether there is a *correct* immediate follow-up action which has lowest distance among *all* actions (or second-lowest distance in the case that the second-last action has lowest distance to the last executed action), which equates to the strongest association between these actions. We call this a *“Correct Most-Probable Action*” (CMPA), being aware that there may be more than one CMPA in any situation. If there are no CMPAs in a current situation then it is probable that the person will either choose an (incorrect) action with stronger association or not know how to proceed, i.e. assistance is required. The concept of assuming that exactly those chunks which have the highest activation (≙ lowest distance) are always chosen is very straightforward and may seem highly simplified given the noisy nature of human behavior. Nonetheless it constitutes a promising heuristic; e.g. it has successfully been used as a basic assumption for a computational cognitive model of instance-based learning [44].

To formalize this approach, let n∈N be the total number of actions related to the considered task and *A* = {*a*_1_, …, *a*_*n*_} the set of all these actions. Let *S* ⊂ *A* be the set of all actions a specific person has already executed in a given situation, including action *a*_*i*_ ∈ *S* as the second-most recent one and *a*_*j*_ ∈ *S* being the most recent one. Let *C*_*S*_ ⊆ *A*\*S* be the set of all correct immediate follow-up actions in this situation, and $D_{a_{x},a_{y}}$ the distance between any two actions *a*_*x*_ and action *a*_*y*_ in the person’s memory (as calculated by SDA-M; see paragraph 2.2.1). Then the value of competent(*S*) indicates whether in this situation, after action *a*_*j*_, the person is assumed to know what to do next on their own:

$$\text{competent}\left(S\right)≔\{\begin{array}{cc} 1 & \text{if}\;\exists a_{c}\in C_{S}:\forall \left(x\in N\;|\;x\leq n\wedge x\neq i,j\right):D_{a_{j},a_{c}}\leq D_{a_{j},a_{x}}\\ 0 & \text{otherwise} \end{array}$$ (1)

In this formula, action *a*_*c*_ is a CMPA. Note that it is not required that there is a correct action with strictly smaller distance than all other actions, but only that it is *among* those actions closest to the most recent one. Action *a*_*j*_ itself as well as its immediate predecessor *a*_*i*_ are hereby disregarded (in contrast to less recent actions from set *S*). Since SDA-M’s pairwise distance values are undirected, it would be neither unexpected nor detrimental to task execution if *a*_*i*_ had lower distance to *a*_*j*_ than all correct follow-up actions, but it seems rather improbable that *a*_*i*_ would be repeated after *a*_*j*_. With respect to these aspects, AMPA is an *optimistic* heuristic.

As an example, assume that exactly these two action sequences are correct for some task:

$$\begin{array}{c} \begin{array}{cc} {\text{seq}}_{1} & ≔(a_{1},a_{2},a_{3},a_{4},a_{7})\\ \text{and}\\ {\text{seq}}_{2} & ≔(a_{1},a_{2},a_{3},a_{5},a_{6}). \end{array} \end{array}$$ (2)

Now assume that a person has already executed the actions (*a*_1_, *a*_2_, *a*_3_) with *S* being the set of this tuple’s elements. If, among all actions, the most recently executed action *a*_3_ has lowest distance to its predecessor *a*_2_, then action *a*_3_ must have *second-lowest* distance to either action *a*_4_ ∈ *C*_*S*_ or action *a*_5_ ∈ *C*_*S*_ for the person to be considered “competent” in this situation. If not, action *a*_3_ must have *lowest* distance to *a*_4_ or *a*_5_. Otherwise the person would be deemed unable to determine a correct follow-up action. For example, if *a*_6_ is closest to *a*_3_ in memory, i.e. ${\text{arg}\;\text{min}}_{a_{x}}\left(D_{a_{3},a_{x}}\right)=a_{6}$, the person would probably try to execute action *a*_6_ after action *a*_3_, which would be wrong.

### 3.3 Algorithm II: Correct Action Selection Probability Analysis (CASPA)

In contrast to the AMPA algorithm, CASPA does not only output a plain binary assessment of competence in a given situation, but a continuous measure of probability. This allows for a much more fine-grained assessment of mental representation structures as well as task-, user- and context-specific thresholds for when to provide assistance. For this purpose CASPA inherited concepts used by the “Adaptive control of thought—rational” (ACT-R) cognitive architecture [45, 46]. According to the ACT-R theory, human behavior is predominately controlled by a central production rule system which is neurophysiologically associated to the basal ganglia. Functionally it is related to procedural knowledge as it represents possible actions as production rules, i.e. “IF—THEN” rules. These rules take current goals, sensory inputs and chunks from declarative memory into account by matching the left side of rules (“IF”) with the contents of buffers associated with the respective subsystems (called “modules”). The right sides of rules (“THEN”) describe possible actions. Overall this symbolic level describes which actions are in principle applicable in a given situation. ACT-R then draws on an additional subsymbolic layer to decide which of the applicable actions shall be executed. This subsymbolic layer is a lower-level abstraction related to neural processes. A very similar mechanism is used for selecting one of several chunks from declarative memory when a specific type of long-term memory content is required. Therefore it does not matter for our purposes whether the actions of a specific task covered by an SDA-M procedure are (in terms of ACT-R) more related to contents of declarative memory or to executive functions associated with the production rule system. In fact, the distinct behavior of the subsymbolic levels of these two processes is modeled using the same basic mathematical approach: The ACT-R mechanisms for selecting production rules and for selecting memory chunks both use the Boltzmann distribution as a “softmax rule” for conflict resolution when more than one rule or chunk is applicable [47]. As we will show now, this approach can be adapted to estimate the probability of a specific person choosing a correct action in a given situation based on SDA-M data.

#### 3.3.1 Calculations

Let *A* = {*a*_1_, …, *a*_*n*_} be the set of all actions related to the considered activity, and *S* ⊂ *A* the set of all actions the person has already executed in a given situation, including action *a*_*i*_ ∈ *S* as the second-most recent and *a*_*j*_ ∈ *S* as the most recent one. Let *C*_*S*_ ⊆ *A*\*S* be the set of all correct immediate follow-up actions in this situation, and *I*_*S*_ ⊆ *A*\*C*_*S*_ be all actions which are *applicable, but incorrect* in the given situation with respect to successful task execution. Then the probability that the person will know what to do after action *a*_*j*_ is estimated as follows:

$$P_{S}=\sum_{a_{c}\in C_{S}}\frac{e^{\rho (a_{j},a_{c})/s}}{\sum_{a_{x}\in \left(C_{S}\cup I_{S}\right)\backslash \left\{a_{i},a_{j}\right\}}e^{\rho (a_{j},a_{x})/s}}$$ (3)

This calculation incorporates a constant *s* > 0 that reflects noise and for our application is set at 0.4, which is a typical value concerning chunk activation in ACT-R [46]. This noise value *s* plays an analogous role to the “temperature” value in Boltzmann machines or simulated annealing [48]: The higher *s*, the less preference is given to actions with higher activation. Eq (3) further requires a measure *ρ*(*a*_*x*_, *a*_*y*_) representing the strength of association between actions *a*_*x*_ and *a*_*y*_ in users’ memory or, in this context equivalently, the activation level of an action *a*_*y*_ after action *a*_*x*_ has been executed. Lander proposed such a measure, called *π*, as part of the original SDA method [36], the predecessor of SDA-M:

$$\pi \left(a_{x},a_{y}\right)=\text{exp}\left(-\frac{D_{a_{x},a_{y}}}{D_{krit}}\right)=1/\text{exp}\left(\frac{\sqrt{1-r_{a_{x},a_{y}}}}{\sqrt{1-r_{krit}}}\right),0<\pi \left(a_{x},a_{y}\right)\leq 1$$ (4)

**Fig 2:**
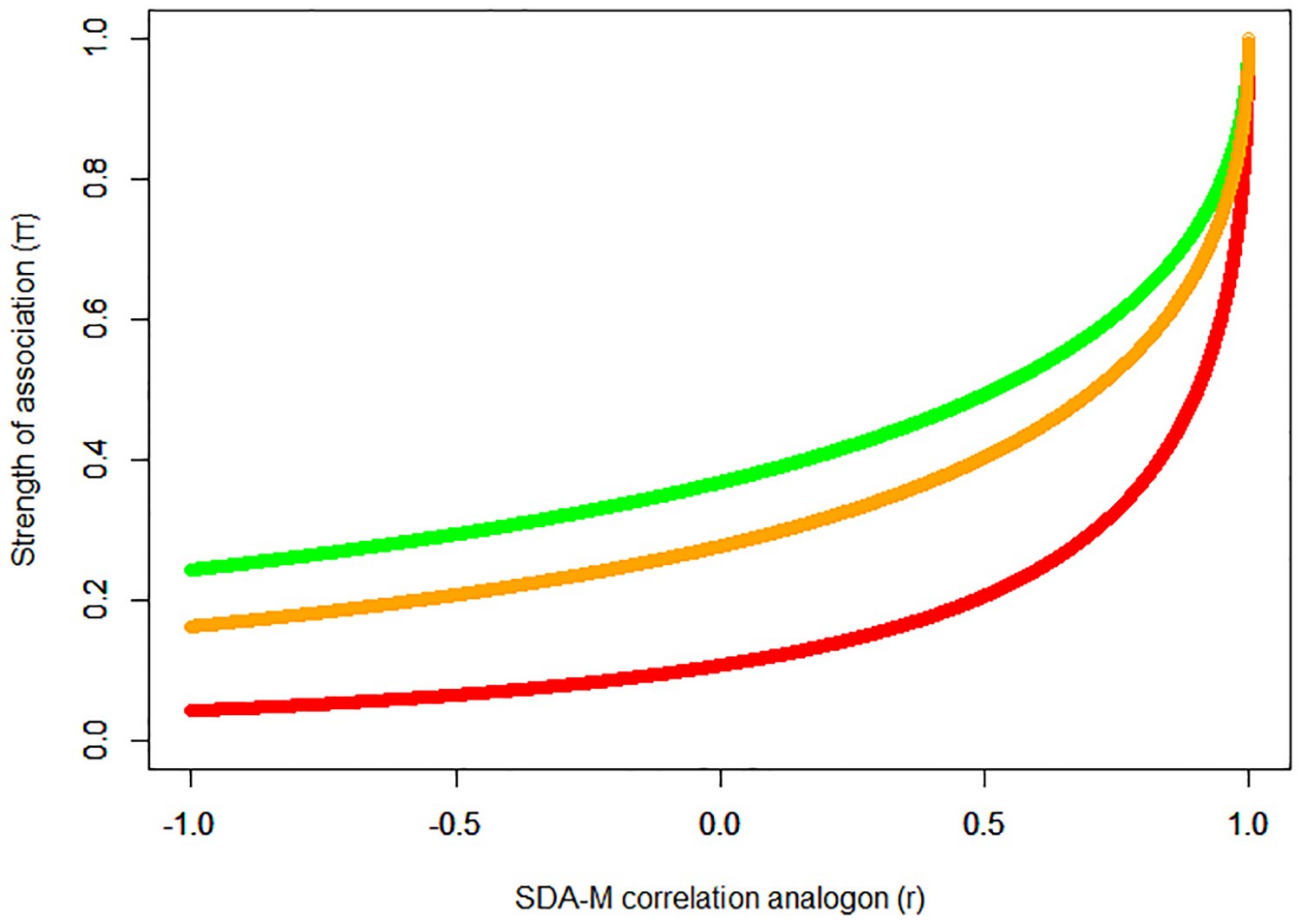
Relation of Lander’s association strength measure *π* to the SDA-M correlation analogon *r*. Green: *r*_*krit*_ = 0. Orange: *r*_*krit*_ = 0.39. Red: *r*_*krit*_ = 0.8.

**Fig 3:**
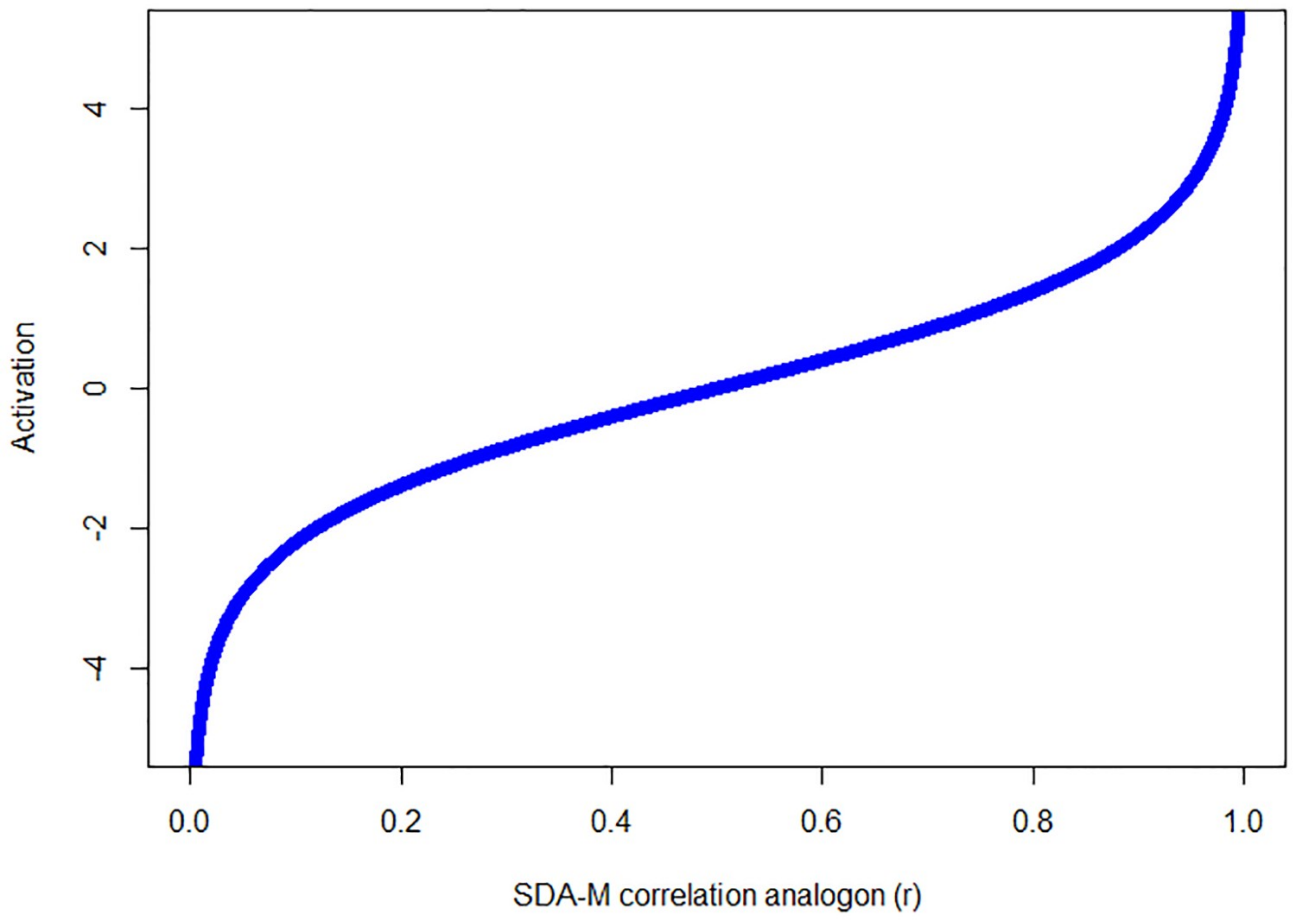
Relation of activation measure *ρ* to the SDA-M correlation analogon *r*.

A drawback of this formula is that the value of *π* depends on the “incidental correlation value” *r*_*krit*_ as defined by [5], which in turn depends on an arbitrarily chosen significance level *α* as well as the total number of actions. Furthermore, uncorrelated and even negatively correlated actions (i.e. $r_{a_{x},a_{y}}\leq 0$) still show association strength values *π*(*a*_*x*_, *a*_*y*_)>0, no matter which *r*_*krit*_ value is determined (cf. Fig 2). Overall the slope of the function leads to insufficient discrimination between negatively or weakly correlated items and moderately correlated ones. To mitigate these issues, an alternative calculation based on the ACT-R formulas for production strength and chunk activation can be used. These formulas reflect the log-odds that an applicable chunk will be matched in the present context [45, 49, 50]. For our purposes, the SDA-M correlation analogon *r* is used analogous to the respective probability values in ACT-R (cf. Fig 3):

$$\text{Activation}\;\rho \left(a_{x},a_{y}\right)≔\text{log}\left(\frac{r_{a_{x},a_{y}}}{1-r_{a_{x},a_{y}}}\right)$$ (5)

Finally, Eq (3) is adjusted to take those actions into consideration which are positively correlated to (i.e. associated with) the most recent action *a*_*j*_, such that CASPA regards these as applicable to the given situation in terms of the ACT-R theory:

$$P_{S}=\sum_{a_{c}\in C_{S},\;r_{a_{j},a_{c}}>0}\frac{e^{\rho (a_{j},a_{c})/s}}{\sum_{a_{x}\in A\backslash \left\{a_{i},a_{j}\right\},\;r_{a_{j},a_{x}}>0}e^{\rho (a_{j},a_{x})/s}}$$ (6)

#### 3.3.2 Default vs. informed threshold

In order to decide whether assistance should be given, arbitrary thresholds for *P*_*S*_ can be used. The most natural approach a priori would be to choose a probability threshold of 0.5, i.e. whether it is supposedly more likely that assistance is needed or dispensable. However, it may be beneficial to determine an *informed threshold* setting using empirical data if available. To this end, a sufficiently large number of SDA-M data sets for the respective task must be available so that the average probability estimated for different situations by CASPA sufficiently converges. The threshold is then set to that average estimated value of *P*_*S*_. The assumed benefit of this is the compensation of possible systematic biases of *P*_*S*_ as determined by CASPA. Such systematic biases may occur when the SDA-M split procedure is slightly easier or harder for subjects to perform than the real task due to artifacts of modeling the real actions in the form of visual and/or textual representations. This approach also takes another potential issue into account: Theoretically, the value of *P*_*S*_ should to some degree be dependent on the total number of actions considered during the SDA-M split procedure. Assuming purely random decisions on part of the subject, it holds that the more actions are included in the split procedure, the lower the expected value of *P*_*S*_. In practice subjects may induce such bias through random tie-breaking in case of doubt as well. Concerning the final binary decision regarding competence or feedback, an empirically informed threshold may mitigate these issues. In the following the binarized output of CASPA using the default threshold (0.5) will be referred to as CASPA_*d*_ while an informed threshold will be denoted as CASPA_*i*_.

### 3.4 Relations between the algorithms

In the following, let the sets *C*_*S*_ and *I*_*S*_ contain only “applicable” actions which are positively correlated with (i.e. associated to) the most recent action *a*_*j*_, i.e. exactly those considered by CASPA (cf. Eq 6). In some special cases the output of CASPA is identical to that of AMPA:

- All applicable follow-up actions are correct:
  |*C*_*S*_| ≥ 1 ∧ *I* = ∅ ⇒ output(AMPA) = output(CASPA) = 1.
- There is no correct applicable action:
  *C*_*S*_ = ∅ ⇒ output(AMPA) = output(CASPA) = 0.
- The noise value is set at *s* → 0 and among the applicable actions with maximum activation is no incorrect action, but at least one correct:
$\left(\forall a_{i}\in I_{S}:\rho \left(a_{j},a_{i}\right)<{\text{max}}_{a_{x}}\rho \left(a_{j},a_{x}\right)\right)\wedge \left(\exists a_{c}\in C_{S}:{\text{max}}_{a_{x}}\rho \left(a_{j},a_{x}\right)=\rho \left(a_{j},a_{c}\right)\right)$ ⇒ output(AMPA) = output(CASPA) = 1
- The noise value is set at *s* → 0 and among the actions with maximum activation is no correct one:
$∄a_{c}\in C_{S}:\rho \left(a_{j},a_{c}\right)={\text{max}}_{a_{x}}\rho \left(a_{j},a_{x}\right)$ ⇒ output(AMPA) = output(CASPA) = 0

It should be remarked that since the regular noise value for CASPA is constant at *s* = 0.4 the relations depending on zero noise are merely theoretical statements. Generally the following relations hold:

- If there is a correct applicable action with maximum activation:
$\exists a_{c}\in C_{S}:\rho \left(a_{j},a_{c}\right)={\text{max}}_{a_{x}}\rho \left(a_{j},a_{x}\right)$ ⇒ 0 < output(CASPA) ≤ output(AMPA) = 1.
- If there are correct applicable actions, but none of these has maximum activation:
$C_{S}\neq \emptyset \wedge \forall a_{c}\in C_{S}:\rho \left(a_{j},a_{c}\right)<{\text{max}}_{a_{x}}\rho \left(a_{j},a_{x}\right)$ ⇒ 0 = output(AMPA) < output(CASPA) <1.

Because of these relations it is not possible to tell in general which algorithm is “more optimistic” or “more pessimistic”, or to derive the output of one algorithm from the other algorithm’s output.

## 4 Evaluation material and methods

As mentioned before, previous studies have demonstrated that human experts (scholars) could use specific visualizations of mental representation structures based on SDA-M data to detect individual issues regarding action execution and derive helpful advice or training concepts for performance optimization. This substantiates the assumption that feasible algorithms would achieve the same if they interpreted SDA-M data in a way that conforms to interpretation by humans. This study investigates to which degree the different computational approaches from section 3 satisfy this criterion.

### 4.1 Data base

In order to establish a suitable test set of SDA-M data as a data pool for further analyses, we cooperated with a local diaconal non-profit foundation working with people with various mental disorders. In a first step, we identified the relevant working tasks related to preparing, opening and cleaning a kiosk at the foundation by observing the operational procedure. These tasks had been used by the foundation as part of an educational program for people with mental disorders for several years. In the second step we interviewed two coaches to detect the underlying working structure. In the third step the amount of working steps was reduced by integrating similar and related steps. In the next step we tested the set of concepts in a pilot study. At the end, the set of working tasks was adjusted and retested. Afterwards, these items were applied to the SDA-M software. A total of 27 trainees with mental disorders, comprising depression, schizophrenia, substance use disorders, autism spectrum disorders, attention deficit hyperactivity disorder, anxiety and mood disorders, used the software to judge whether a pair of actions belongs together during their work in the kiosk. All participants gave informed consent in written form. Their capacity to do so was ensured by asking our contacts at the foundation (trained professionals in coaching people with disabilities) to exclude all trainees for whom this might be questionable. In the SDA-M splitting procedure, a total of 15 different actions were covered, which could be divided into four independent activities:

*Kiosk preparation*:

- Refill cutlery cart
- Fill in coffee beans and cocoa
- Refill fridge with drinks
- Put plate for rolls in place
- Allocate cart for dirty dishes

*Kiosk customer service*:

1. Welcome the customer and take the order
2. Prepare coffee and cocoa
3. Serve drinks and food
4. Take the money

*Kiosk wrap-up*:

- Wash the dishes and start the dishwasher
- Clean the glass pane of the refrigerator
- Clean the coffee machine
- Wipe the surfaces

*Laundry*:

1. Wash the laundry
2. Hang up and iron the laundry

The actions related to customer service and laundry naturally have to be executed in a sequential order (as indicated by the numbering above). Therefore, ideally these actions should pairwise be strongly associated in long-term memory structures to represent the correct sequence. Actions related to the kiosk preparation and wrap-up activities can be executed in arbitrary order (indicated by bullet points). Generally, actions related to different activities should ideally not be associated to each other in long-term memory.

The trainees were familiar with these actions and activities to differing degrees, because they were trained in these tasks at the diaconal organization for different lengths of time (between a few weeks and several months). In line with previous studies, e.g. on actions in judo [42], windsurfing [16], soccer [51] or manual actions in humans and robots [12, 13], we assume that potential problems and deficits in action execution are reflected in the mental structure of the tasks. Thus, unrelated or wrongly related actions on the cognitive level are expected to lead to decreased real-life performance, e.g. forgetting of the next relevant action or executing a wrong task. For example, a trainee might start cleaning a table instead of serving a customer who is waiting in line.

### 4.2 Retrieval of experts’ manual assessments

The data pool was then used to compare assessment by human SDA-M experts with algorithmic interpretation. To this end, we created an assessment task consisting of 80 different hypothetical situations related to the kiosk-servicing activities listed above. Each of these “situations” was specified by

- SDA-M data visualization of a random subject, and
- a fictitious sequence of actions this subject was said to have executed up to now.

The fictitious action sequences had been created by selecting representative subsets from the set of all correct sequences and applying a random cut-off length to each sequence. By definition, a “correct sequence” was a sequence containing actions from only one of the four activities and, where applicable, in correct temporal order. The first half of the final set of sequences was initially chosen randomly. The resulting set was then manually revised to mitigate a bias towards sequences from the larger, unordered activities caused by the disproportionate number of permutations of actions in these activities. The second half was determined by randomly selecting from a set of sequences that was priorly adjusted by adding duplicates of some sequences to compensate for over-/underrepresentation of activities. The resulting test sequences are provided as S1 Table.

**Fig 4:**
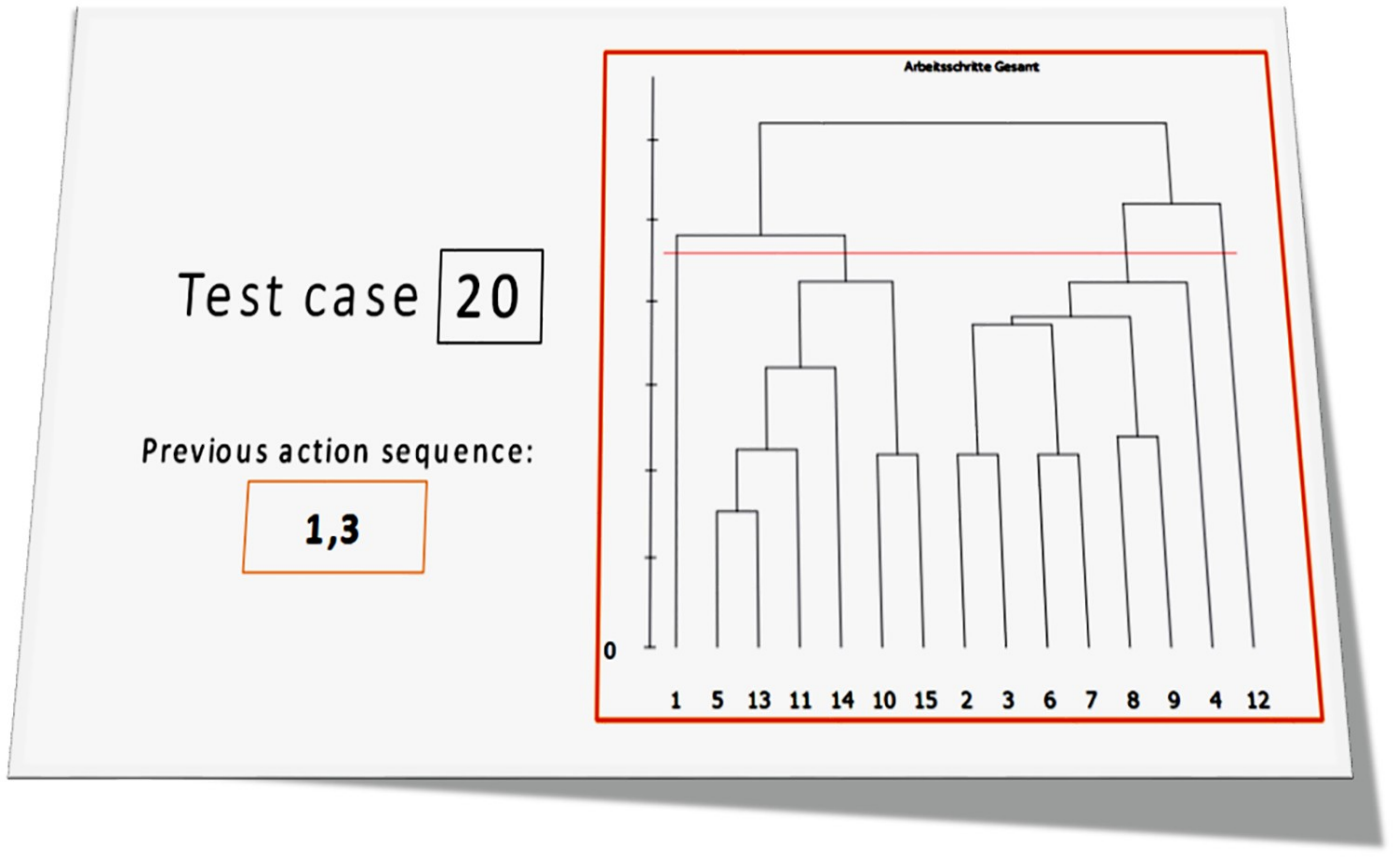
Example of a test case representing a fictitious “situation” for assessment. The right side shows a dendrogram visualizing a subject’s mental representation structure for the kiosk service activities. In this example some actions from the “kiosk preparation” activity (IDs 2, 3 and 4) are clustered with actions from the “customer service” activity (IDs 6, 7, 8 and 9), indicating a corresponding relation in memory.

These “situations” or test cases were then presented (as shown in Fig 4), one after another, to a group of *N* = 12 human scholars, along with a general overview of all correct sequences for each of the four kiosk-service-related activities. The participating scholars were experts with extensive education regarding the SDA-M method and personally experienced in using it for scientific purposes before, but they were blind with respect to the algorithmic analyses that we investigated in this study. Each scholar had to assess independently for each situation, based on the given SDA-M data visualizations, whether or not the respective subject would more likely need assistance or be able to determine a correct follow-up action in the given situation (see S1 File for survey sheets). The same test cases were also fed into our AMPA and CASPA algorithms. As both experts and algorithms pursued the same goal (predicting human errors), their results could then be compared as described in the next section.

## 5 Data analysis and results

The assessments by each of the human experts have been translated into binary vectors with value 1 representing the assessment “*the subject in this scenario is probably able to determine a correct follow-up action in the given situation on their own, i.e. assistance is not required*”, and value 0 representing the opposite case. We found that the assessments from 11 out of 12 human SDA-M experts correlated positively with the group average, while those from one expert correlated negatively. Presumably this was due to misunderstandings regarding the assessment task. Therefore we excluded this expert’s ratings from further analyses. The remaining assessments served as our ground truth for comparison with the respective results from the AMPA and CASPA algorithms. As CASPA delivers estimated probability values *P*_*S*_ ∈ [0, 1], a direct comparison was possible with the portion of experts *P*_*E*_ ∈ [0, 1] who supposed in each test case that the respective subjects were competent. We found a positive correlation of *r* = .62 which, considering that the average correlation (determined using Fisher *z*-transformation) of each individual expert’s assessments to the average assessments of the remaining experts was almost identical ($\bar{r}=.59$), indicates an adequate fit between manual and algorithmic assessments.

In order to evaluate the (binary) output from AMPA and the influence of different thresholds for CASPA, several common metrics for the evaluation of binary classifiers have been employed. For this purpose the Median value of the experts’ assessments was used for each test case. Due to an odd number of experts (*N* = 11) this equals majority decision. CASPA’s continuous *P*_*S*_ values were converted into binary decisions as described in section 3.3.2, i.e. using either the default threshold of 0.5 (“CASPA_*d*_”) or an informed threshold of $\bar{P_{S}}=0.2396$ (“CASPA_*i*_”), where $\bar{P_{S}}$ was the average of all probability values output by CASPA for all 80 test cases from the study. In the following, let *N*_*ab*_ with *a*, *b* ∈ {0, 1} be the total number of situations where the human experts’ assessment equals *a* and an algorithm’s prediction equals *b*. The simple matching coefficient (SMC) for binary vectors yields the percentage of cases where human and algorithmic assessments came to the same results, thus representing the accuracy of matching the human experts’ assessments regarding expected action errors:

$$\text{Accuracy}=\text{SMC}=\frac{N_{00}+N_{11}}{N_{00}+N_{01}+N_{10}+N_{11}}$$ (7)

One-tailed binomial tests with *H*_1_: *P*(success) > *P*(failure) were performed for each algorithmic approach to determine whether the degree of match between human and algorithmic assessments, i.e. the accuracy, is significantly above chance level. Hereby each matching pair of assessments counted as a successful Bernoulli trial and each deviating pair as a failure. Correlations between the respective vectors of binary decisions were calculated and tested for significance as well. Sensitivity, specificity, and positive/negative predictive values can be defined analogously to accuracy (Eqs 8 to 11). In this context a “true positive” denotes cases of both algorithm and human experts suspecting that assistance was required because the subject’s mental representation structure is not suitable (*N*_00_).

$$\text{Sensitivity}=\frac{N_{00}}{N_{00}+N_{01}}$$ (8)

$$\text{Specificity}=\frac{N_{11}}{N_{11}+N_{10}}$$ (9)

$$\text{Positive}\;\text{predictive}\;\text{value}\;\text{(PPV)}=\frac{N_{00}}{N_{00}+N_{10}}$$ (10)

$$\text{Negative}\;\text{predictive}\;\text{value}\;\text{(NPV)}=\frac{N_{11}}{N_{11}+N_{01}}$$ (11)

In addition to these classic metrics the *balanced accuracy* should be considered, because this measure safeguards against a biased classifier taking advantage of an imbalanced test set [52]. If an algorithm performs equally well regarding its positive and negative predictive values, its balanced accuracy reduces to the conventional accuracy.

$$\text{Balanced}\;\text{accuracy}=\frac{1}{2}\left(\frac{N_{11}}{N_{11}+N_{01}}+\frac{N_{00}}{N_{00}+N_{10}}\right)$$ (12)

**Fig 5:**
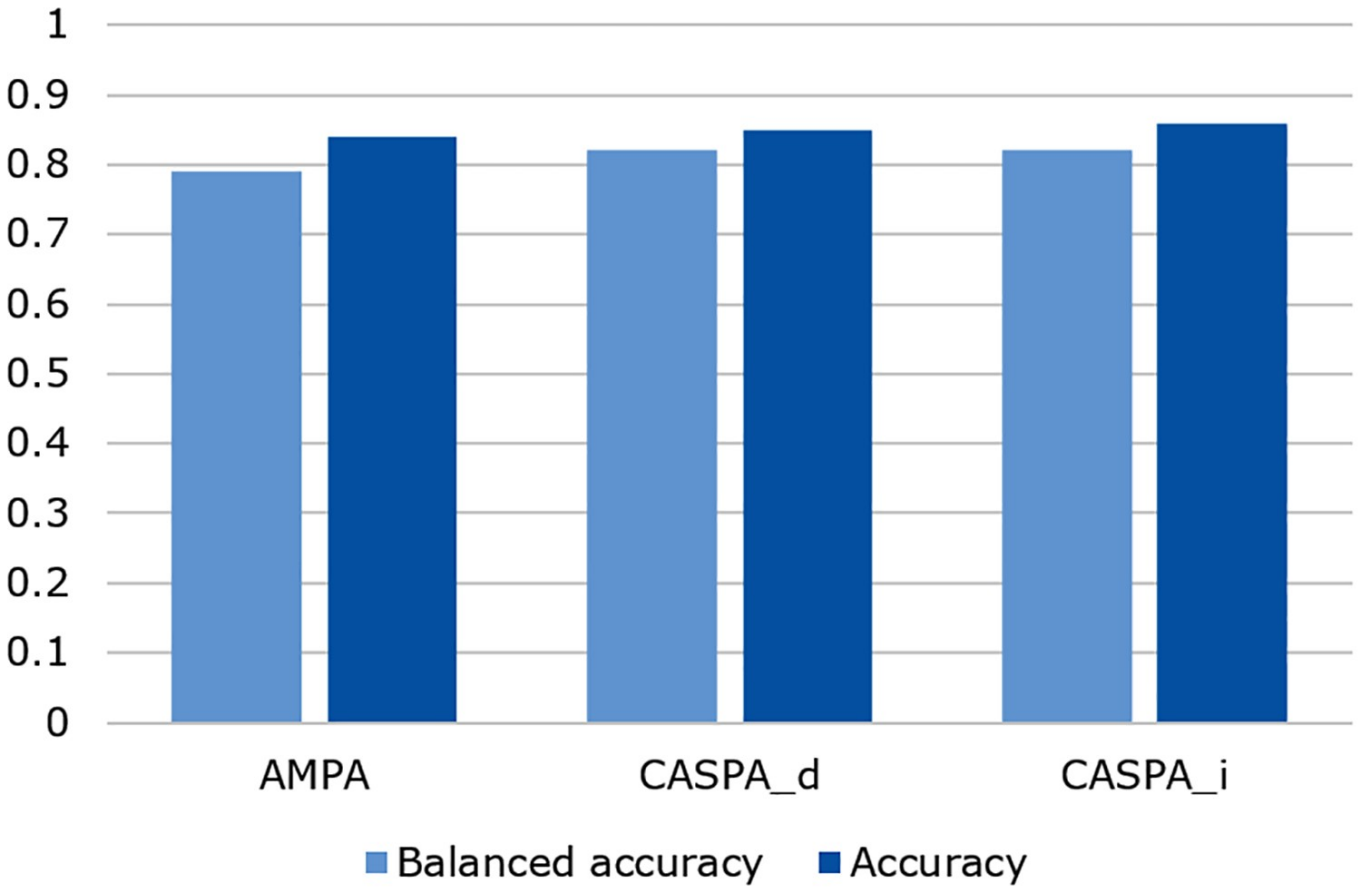
Key results of the evaluation study. Accuracy and balanced accuracy (see Eq 12) measure the congruence of algorithmic (AMPA, CASPA_*d*_, CASPA_*i*_) and human experts’ assessments.

Table 1 shows how AMPA, CASPA_*d*_ (threshold = 0.5), and CASPA_*i*_ (threshold = 0.2396) performed with respect to these metrics. Balanced and conventional accuracy values were close to each other with all algorithms ranging between 0.79 and 0.86 for these metrics (Fig 5). Binomial tests showed that with all three algorithm variants the match with human experts’ assessments was highly significant above chance level. Differences between the algorithms were marginal, though CASPA_*i*_ generally tended to score slightly better than AMPA.

**Table 1:** Full results of the evaluation study.

Algorithm	Correlation	Accuracy	Sensitivity	Specificity	PPV	NPV	Balanced accuracy
AMPA	0.61***	0.84***	0.86	0.77	0.91	0.68	0.79
CASPA_*d*_	0.61***	0.85***	0.93	0.64	0.87	0.78	0.82
CASPA_*i*_	0.67***	0.86***	0.88	0.82	0.93	0.72	0.82

*** *p* < 0.0001.

## 6 Discussion

The analysis of mental representation structures using the SDA-M method is a well-established approach (cf. e.g. [5, 6, 12, 13, 25, 27–32, 34, 35]) for gaining insight into the degree of individual expertise related to various activities, ranging from basic grasping actions to complex system interactions. Traditionally this information was computationally pre-processed and visualized to be interpreted by human SDA-M experts. As this requires human resources, specific training and is time-demanding, this approach is inefficient, nondeterministic and not applicable in real-time systems. Therefore we investigated different approaches to algorithmically automatize the interpretation of SDA-M data. In order to enable suitable predictions about error-prone steps during task execution, specific prerequisites must be satisfied. Most notably, the considered activity must be divisible into a limited set of sequential actions or sub-tasks which can be assumed to be executable without issues. When used as a component of a technical assistance system, this approach is most advantageous if the expected benefits from error predictions outweigh spending approximately 10-15 minutes for performing the SDA-M split procedure before system usage. This may commonly apply when executing the assisted actions in reality is relatively time-consuming and/or when errors have severe consequences, e.g. when wrong actions are difficult to reverse. Presumably it might also help specific target groups overcoming insecurity and hesitation to tackle unfamiliar activities. In order to take learning processes into account and further reduce unneeded assistance, users may want to update the data about their mental representation structures from time to time by repeating the SDA-M split procedure.

In a first evaluation study, our proposed algorithms for SDA-M-based error prediction, AMPA, CASPA_*d*_ and CASPA_*i*_, showed a high degree of consistency with human experts’ assessments about probable action errors based on SDA-M visualizations of subjects’ mental representation structures. The percentage of matches between algorithmic and experts’ assessments was significantly higher than would be expected by chance, ranging from 84% to 86%. The differences between the proposed algorithmic variants were insignificant, but the more sophisticated CASPA_*i*_ algorithm scored slightly higher regarding all metrics we considered than the simpler AMPA algorithm. It should be noted that the existence of some non-matching cases did not necessarily imply that the respective algorithmic predictions were wrong. On the one hand, human experts also varied from one another in their judgments regarding error predictions to some degree. On the other hand, some of the information contained in the raw data is lost when visualizing mental representation structures via dendrograms for manual interpretation. On this account the algorithmic interpretations may actually have been better than those from human experts. However, due to a lack of definitive ground truth regarding the actual mental structures of subjects from this study, this hypothesis can neither be confirmed nor rejected so far. Generally, the evaluation study reported in this article constitutes a proper indication of suitability of the algorithmic interpretations of SDA-M data in comparison with the traditional approach of manual assessment for a specific task. Noteworthy limitations of the study are the relatively small number of activities that were analyzed, as well as the present empiric evidence in favor of our new algorithmic approaches being restricted to a comparison with experts’ assessments.

## 7 Outlook

Further research is mandatory to reliably assess the degree of match between predicted errors and human errors actually occurring during task execution in reality. Pertaining to categorizations of human errors [53, 54], we expect our approach to cover most (knowledge- and rule-based) mistakes, and potentially also some types of slips, e.g. due to associative activation and capture errors (excluding external event sources), loss of activation and faulty triggering. However, since many occurrences of slips are context-dependent and unreproducible, the SDA-M split procedure certainly cannot be expected to capture all (and possibly not even most) instances of slips. A promising extension may be to incorporate eyetracking and physiological sensor measurements in addition to SDA-M data into a real-time error prediction component. This data can be used to determine users’ current stress level, which in many situations influences the predetermined probability of making errors.

An interesting philosophical question is under which conditions assistance systems incorporating our prediction module may be considered *anticipatory systems* according to Rosen [55]. Following Rosen’s pertinent definition, this would be the case if the human user is regarded as (part of) the system’s environment, and the system’s internal predictive model “provides an alternate description of the entailment structure of the mapping representing the [biological] process itself” [56]. With respect to the intended application this seems to be the case if (and only if) the predictive model is grounded on neurocognitive actualities. Arguably, both Schack’s theory (CAA-A) [24] underlying the SDA-M method and Anderson’s ACT-R, from which CASPA’s calculations are derived, may be regarded as sufficiently well-grounded in this regard. Furthermore, the predictive model *M* of an anticipatory system *S* should be “equipped with a set *E* of effectors that operate either on *S* itself or on the environmental inputs to *S*, in such a way as to change the dynamical properties of *S*” [56]. Such effectors could for example be the visual or auditory displays of an assistance system which cause its user to behave in a different way, i.e. “the effect of the model *M* creates a discrepancy—*S* would have behaved differently if *M* were absent” [56]. According to Louie [56], such a “predictive or anticipatory mode” would cause a system to “become more like an organism, and less like a machine”.

## Supporting information

S1 Table Test sequences list. Spreadsheet table containing the action sequences used as test cases (two blocks with 40 test sequences each).
(ODS)

S1 File SDA-M experts assessments survey sheets. ZIP file containing the response sheets that were provided to each SDA-M expert for indicating their assessment of each test case.
(ZIP)
