# Correction: Computational assessment of long-term memory structures from SDA-M related to action sequences

**DOI:** 10.1371/journal.pone.0214189

**Published:** 2019-03-18

**Authors:** The PLOS ONE Staff

In the 3.3.1 Calculations subsection, there are errors in Eqs (3) and (6); a set difference operator (∖) was incorrectly removed from both equations during the typesetting process. The publisher apologizes for these errors. Please view the complete, correct Eq (3) and Eq (6) here:

$$P_{S}=\sum_{a_{c}\in C_{S}}\frac{e^{\rho \left(a_{j},a_{c}\right)/s}}{\sum_{a_{x}\in \left(C_{S}\cup I_{S}\right)\backslash \{a_{i},a_{j}\}}e^{\rho (a_{j},a_{x})/s}}$$ (3)

$$P_{S}=\sum_{a_{c}\in C_{S},r_{a_{j},a_{c}}>0}\frac{e^{\rho \left(a_{j},a_{c}\right)/s}}{\sum_{a_{x}\in A\backslash \{a_{i},a_{j}\},r_{a_{j},a_{x}}>0}e^{\rho (a_{j},a_{x})/s}}$$ (6)
