# Supplementary material for: Computational assessment of long-term memory structures from SDA-M related to action sequences
Source: PLoS One. 2019 Feb 22;14(2):e0212414. doi: 10.1371/journal.pone.0212414 (PMC6386273; doi:10.1371/journal.pone.0212414)
Supplement: S1 File — ZIP file containing the response sheets that were provided to each SDA-M expert for indicating their assessment of each test case. (ZIP) [file pone.0212414.s002.zip › Questionnaire_Block_1.pdf]

Test Block 1 Name:

| Test case | Assistance needed regarding next action? | |
|---|---|---|
| **1** | yes □ | no □ |
| **2** | yes □ | no □ |
| **3** | yes □ | no □ |
| **4** | yes □ | no □ |
| **5** | yes □ | no □ |
| **6** | yes □ | no □ |
| **7** | yes □ | no □ |
| **8** | yes □ | no □ |
| **9** | yes □ | no □ |
| **10** | yes □ | no □ |
| **11** | yes □ | no □ |
| **12** | yes □ | no □ |
| **13** | yes □ | no □ |
| **14** | yes □ | no □ |
| **15** | yes □ | no □ |
| **16** | yes □ | no □ |
| **17** | yes □ | no □ |
| **18** | yes □ | no □ |
| **19** | yes □ | no □ |
| **20** | yes □ | no □ |
| **21** | yes □ | no □ |
| **22** | yes □ | no □ |
| **23** | yes □ | no □ |
| **24** | yes □ | no □ |
| **25** | yes □ | no □ |
| **26** | yes □ | no □ |
| **27** | yes □ | no □ |
| **28** | yes □ | no □ |
| **29** | yes □ | no □ |
| **30** | yes □ | no □ |
| **31** | yes □ | no □ |
| **32** | yes □ | no □ |
| **33** | yes □ | no □ |
| **34** | yes □ | no □ |
| **35** | yes □ | no □ |
| **36** | yes □ | no □ |
| **37** | yes □ | no □ |
| **38** | yes □ | no □ |
| **39** | yes □ | no □ |
| **40** | yes □ | no □ |
